# Parallel Time-Frequency Multi-Scale Attention with Dynamic Convolution for Environmental Sound Classification

**DOI:** 10.3390/e27101007

**Published:** 2025-09-26

**Authors:** Hongjie Wan, Hailei He, Yuying Li

**Affiliations:** Information Engineering Department, Beijing University of Chemical Technology, No. 15 North Third Ring Road East, Beijing 100029, China

**Keywords:** multi-scale convolution, CNN, dynamic convolution, environmental sound classification, deep learning

## Abstract

Convolutional neural network (CNN) models are widely used for environmental sound classification (ESC). However, 2-D convolutions assume translation invariance along both time and frequency axes, while in practice the frequency dimension is not shift-invariant. Additionally, single-scale convolutions limit the receptive field, leading to incomplete feature representation. To address these issues, we introduce a parallel time-frequency multi-scale attention (PTFMSA) module that integrates local and global attention across multiple scales to improve dynamic convolution in order to overcome these problems. We also introduce the parallel branch structure to avoid mutual interference of information in case of extracting time and frequency domain features. Additionally, we utilize learnable parameters that can dynamically adjust the weights of different branches during network training. Building on this module, we develop PTFMSAN, a compact network that processes raw waveforms directly for ESC. To further strengthen learning, between-class (BC) training is applied. Experiments on the ESC-50 dataset show that PTFMSAN outperforms the baseline model, achieving a classification accuracy of 90%, competitive among CNN-based networks. We also performed ablation experiments to verify the effectiveness of each module.

## 1. Introduction

The rapid development of information technology has led to a flood of sound signals in daily life, and each carries valuable information about the physical environment [1]. Consequently, sound signals contain a great deal of information, and their processing facilitates our understanding of specific physical situations. Among them, environmental sound classification (ESC) plays a vital role, and the goal is to classify the environment based on surrounding environmental sounds [2]. ESC covers a wide range of topics and a wide range of categories, such as animals, natural soundscapes, humans, domestic sounds, urban noises, etc. ESC is also used in a wide range of practical applications such as surveillance systems [3,4], machine hearing [5,6], environmental monitoring [7], crime alert systems [8], soundscape assessment [9], smart cities [10], etc. Because ESC aims to classify acoustic signals in complex environments where a variety of unknown sound sources are present, it remains an open research topic with a wide range of applications. We work on the ESC task and aim to propose a network to better carry out the classification task.

Due to the rapid rise of deep learning (DL) and its success in other fields [11], such as biomedical waste management [12], real-time agricultural disease diagnosis [13], and speech enhancement [14], a large number of deep learning models have been widely used for ESC tasks in recent years [15,16,17,18,19,20,21]. However, environmental sounds are dynamic and unstructured, which makes it more difficult to design appropriate feature extraction networks. We improved the model and conducted extensive experimental comparisons to address this issue, ultimately proposing a compact architecture with small parameters but high classification accuracy. In this study, we made the following key contributions:

(1) An improved dynamic convolution that extracts dynamic attention by combining a convolution and a fully connected layer. This approach not only ensures global and local correlation but also maintains the shift-invariance characteristic of the 2D convolution in the frequency dimension.

(2) Frequency multi-scale attention (FMSA) and temporal multi-scale attention (TMSA) modules are proposed, which apply improved dynamic convolution for multi-scale feature extraction in frequency and time dimensions, respectively. This enables the features to contain more information to avoid the limitations of the convolutional feature and ensures physical consistency.

(3) The PTFMSA module is used to extract temporal and frequency features from audio. It adopts a parallel branching structure to avoid interference between features of different dimensions and learns adjustable dynamic parameters to adjust the weights of different branches during network training.

(4) As previously described, our novel PTFMSAN architecture for ESC applies the PTFMSA module and convolution layer to directly process raw audio input. Experimental results on the ESC-50 dataset show that PTFMSAN performs better than the baseline network, achieving a classification accuracy of 90%, which is higher than other state-of-the-art CNN-based networks.

The remaining part of the paper is organized as follows. Section 2 describes the modules and network we propose. Section 3 describes the proposed methodology. Section 4 shows the experimental work. The result and discussion are given in Section 5. Section 6 represents the conclusion.

## 2. Related Research

Through extensive experiments, people have discovered that convolutional neural networks (CNNs) can provide the most advanced performance compared to other structures [22,23]. Initially, CNN was used for feature extraction in the ImageNet [24] competition to perform convolution on 2D features. However, the raw audio signal is a 1D signal. In order to apply CNN in audio feature extraction, the signal is converted into a 2D spectrogram. Therefore, spectrograms and mel-scale frequency cepstral coefficients (MFCC) are often used as inputs to the network. For example, Piczak [25] proposes two convolutional layers in tandem with two fully connected layers to evaluate the potential of CNNs in environmental classification. The results show that the classification accuracy of the proposed network is better than other state-of-the-art methods. Behnaz et al. [26] proposed a recurrent neural network (RNN) coupled with CNN to solve the problem, but the network needs a large dataset to show its efficient performance. The experimental results show that the proposed CNN-RNN architecture model achieves better performance.

**Figure 1:**
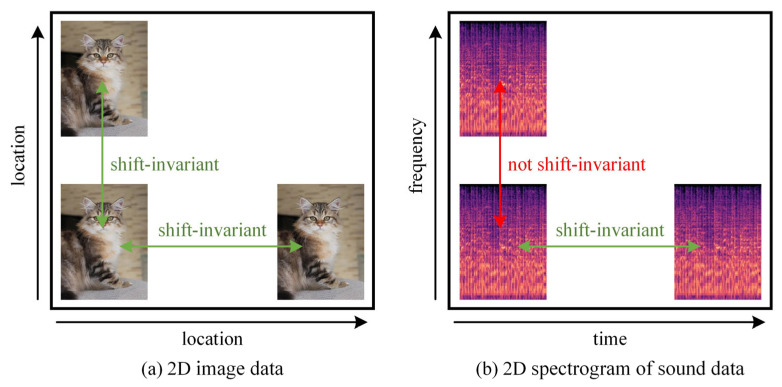
The illustration of shift-invariance.

Two-dimensional convolution has been widely used in feature extraction of spectrograms of sound signals and has achieved certain success. However, 2D convolution has been proposed to recognize 2D image data and is therefore not fully compatible with audio data. Figure 1 illustrates the different shift-invariance of 2D image data and 2D audio data. Since shifting 2D image data in two dimensions does not result in a change of information, it only differs in position. Therefore, it remains unchanged in both dimensions, and convolution operations in any dimension are physically continuous. But the spectrogram of an audio signal sounds different when it is shifted along the frequency axis. This means that the spectrogram of the audio is shift-invariant only along the time axis and not along the frequency axis. To solve this problem, Nam et al. [27] proposed Frequency Dynamic Convolution (FDC), which applies kernels that adapt to the input frequency components. However, extracting attention using convolution instead of fully connection leads to a loss of global associations. Inspired by this, we propose an improved FDC by combining these two structures. In addition, the single scale of FDC makes the receptive field limited, resulting in insufficiently rich local features. Based on the above factors, this paper proposes two modules: an (a) FMSA module and a (b) TMSA module. These two modules apply improved dynamic convolution for feature extraction at multiple scales in the frequency and time dimensions, respectively. This makes the features contain more information to avoid the limitations of the convolutional feature and ensure physical consistency.

In order to obtain rich sound information, features are extracted from both the time and frequency domains. The traditional way of acquiring time-frequency features is to directly cascade two modules [28,29]. However, the cascade approach has the disadvantage that temporal and frequency features may interfere with each other, which will affect the effectiveness of the network. Therefore, we propose the PTFMSA module that uses the structure of parallel branches along the time and frequency dimensions to avoid interference between features of different dimensions. The importance of different branches is different, and if the corresponding weights are fixed, it will make the network ineffective. Therefore, we use learnable parameters that can dynamically adjust the weights of different branches during network training to obtain the optimal coefficients.

After applying the PTFMSA module, we propose a parallel time-frequency multi-scale attention network (PTFMSAN) for ESC tasks. This network applies 1D convolution to replace the conversion from raw audio to spectrograms. It not only allows the raw audio to be used directly as input to the network but also allows the network to dynamically adjust features. In addition, Tokozume et al. [30] proposed a mixing method called between-class (BC) learning, which can increase the number of training datasets during the training phase of deep learning models. The mixing strategy is where sounds between classes are generated by mixing two sounds belonging to different classes at a random rate. At the same time, BC learning also takes the sound pressure level of the raw sound signal into account for balancing accordingly. The study shows that the BC learning approach performs better in sound recognition networks and data enhancement. Therefore, we used BC learning to process the dataset to improve the model. PTFMSAN is evaluated on a benchmark dataset [31] and achieves a state-of-the-art classification accuracy of 90%. We also performed ablation experiments to verify the effectiveness of each module. Due to the small parameter count and high classification accuracy, PTFMSAN can be quickly trained and deployed on real-world devices for practical classification tasks.

Recent Transformer-based ESC models: In addition to CNNs, Transformer-based techniques have recently demonstrated exceptionally high ESC-50 accuracies. With Audio Set pre-training, the Audio Spectrogram Transformer (AST) achieves 95.6% accuracy, HTS-AT reaches 97.0%, and the Efficient Audio Transformer (EAT) achieves approximately 96%. Tens of millions of parameters are frequently required for these models, which typically rely on deep architectures and extensive pre-training. On the other hand, we concentrate on a CNN-based architecture that is small (about 3.7 M parameters) and can be trained directly on ESC-50. As a result, although we acknowledge the improved absolute performance of current Transformer-based models, we mainly compare with CNN baselines.

## 3. The Proposed Method

**Figure 2:**
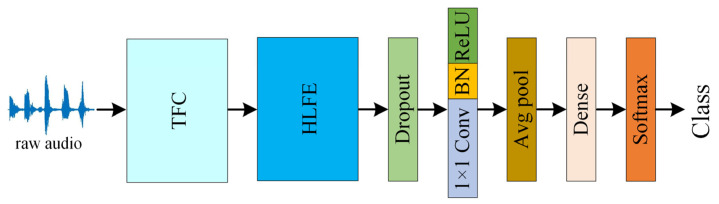
The architecture of PTFMSAN.

This section proposes a parallel time-frequency multi-scale attention network (PTFMSAN) for ESC tasks as shown in Figure 2. Firstly, the raw audio is converted into 2D frequency features through the time-frequency conversion (TFC) module. The high-level feature extraction (HLFE) module then fully extracts time-frequency features. After that, a 2D convolutional layer with a kernel size of 1 and an average pooling (avgpool) layer are used for post-processing. Finally, features are flattened and then fed into the softmax output layer for classification. The following subsections first introduce the TDC and HLFE module. Then, the detailed improved dynamic convolution is shown, which is the core of the PTFMSA module. Next, the detailed PTFMSA module is given.

### 3.1. TFC and HLFE Module

**Figure 3:**
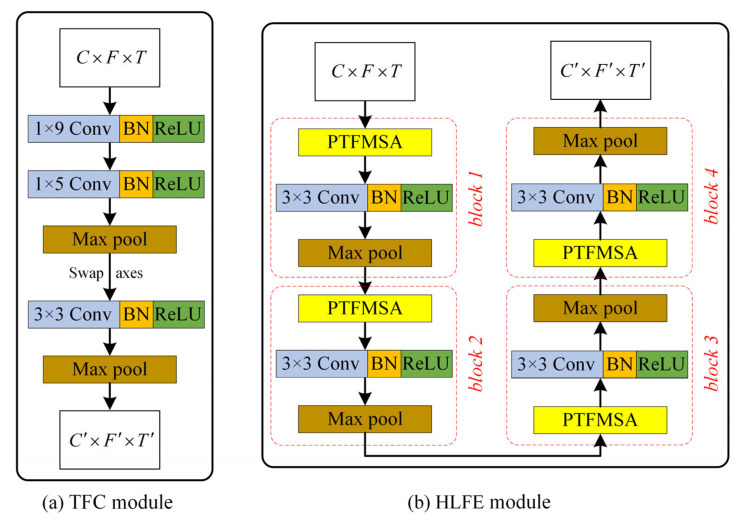
(**a**) Structure of the TFC module, (**b**) structure of the HLFE module.

In order to apply 2D convolution to audio data, it is often necessary to convert the raw audio signal into a 2D spectrogram as a network input. However, it is a very time-consuming and empirical task to select the appropriate frequency domain features and dimensions. The proposed TFC module dynamically performs time-frequency conversion in the network based on convolution, so that raw audio can be directly used as an input to the network. As illustrated in Figure 2, the overall architecture begins with a raw waveform of size 1 × *T*. Figure 3a further details the TFC block: the input waveform (1 × *T*) passes through two 1-D convolution layers (kernel size 9 and 5) with BN and ReLU, followed by max-pooling. The resulting features are then reshaped and swapped to *C* × *F* × *T* representation, which serves as a learned filter bank-like input for subsequent 2-D operations. After this step, a 2D convolution layer with a kernel size of 3 is used to preprocess and then followed by a max-pooling layer.

Effective receptive field: The TFC block’s initial two 1-D convolution kernels are rather small (sizes 9 and 5 at 44.1 kHz), but the stacking of convolution and pooling layers causes the effective receptive field to increase quickly. Consequently, the network can record both transients or short-term stimuli and the longer-term context required for low-frequency data. We note that using larger or dilated kernels at the input stage may enhance low-frequency modeling even more, and we point to this as an interesting avenue for additional research.

After obtaining the 2D features, further time-frequency high-level features need to be extracted. As shown in Figure 3b, the HLFE module mainly consists of 4 blocks. Each block is composed of a PTFMSA layer and a 2D convolutional layer with a kernel size of 3, followed by a maxpool layer. All convolution layers are followed by a BN and a ReLU layer by default. The PTFMSA module extracts multi-scale time-frequency features, and then the level of features is improved by the 2D convolutional layer. Finally, the features are compressed by the maxpool layer. The HLFE module is formed by stacking 4 blocks, which are used to extract high-level features.

### 3.2. Improved Dynamic Convolution

**Figure 4:**
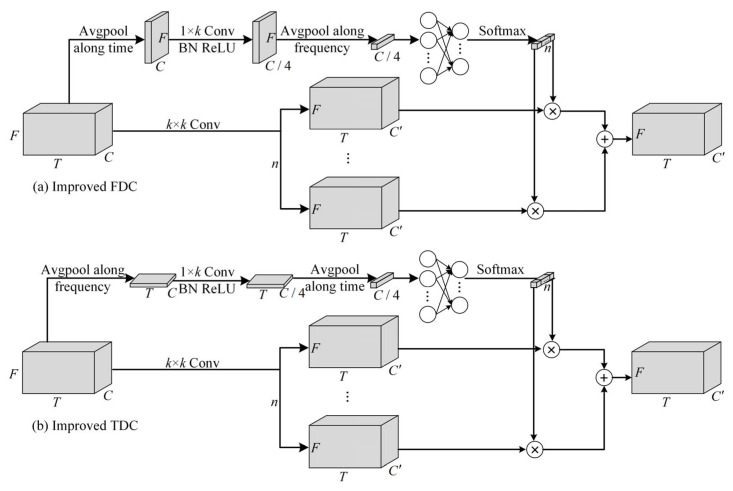
Improved FDC and improved TDC.

As shown in Figure 4, improved FDC and improved temporal dynamic convolution (TDC) are implementations of improved dynamic convolution in the frequency and time domains, respectively. Take improved FDC, for example; it contains two branches, one is the attention branch and the other is the convolutional branch. In the attention branch, input feature map $U\in R^{T\times F\times C}$ is first compressed along the time dimension by avgpool. The 1 × *k* convolution followed by a BN and a ReLU layer is applied to extract frequency local correlation features ${\hat{U}}^{f}\in R^{1\times F\times \frac{C}{4}}$ to ensure shift-invariance of the 2D convolution along the frequency dimension. The reduction from C channels to $\frac{c}{4}$ is achieved through an additional 1 × 1 point wise convolution after the 1 × *k* operation, which serves as a channel projection for efficiency.

(1) $$U^{f}=avg(U,T)$$

(2) $${\hat{U}}^{f}=ReLU\left(BN\left(Conv\left(U^{f}\right)\right)\right)$$

where *avg (•, T)*, *Conv (•)*, *BN (•)*, and *ReLU (•)* denote the avgpool along time, the convolution block, the batch normalization, and the ReLU activation, respectively.

Then, frequency local correlation features ${\hat{U}}^{f}$ are compressed along the frequency dimension by avgpool and the fully connected layer is used to extract global feature attention $A^{f}\in R^{1\times 1\times \frac{C}{4}}$. If only convolution is applied to extract the attention weights, it leads to loss of global features. If only fully connected layer is applied to extract the attention weights, it leads to the 2D convolution that does not satisfy shift-invariance along the frequency dimension. This is why we combine convolutional and fully connected layers for improvement. Next, softmax is performed along the channel dimension to make the attentional weights range from 0 to 1 and make the sum of the weights of the different basis kernels to be 1 ${\bar{A}}^{f}\in R^{1\times 1\times n}$.

(3) $$A^{f}=avg\left({\hat{U}}^{f},\;F\right)$$

(4) $${\bar{A}}^{f}=S\left(FC\left(A^{f}\right)\right)$$

where *avg (•, F)*, *FC (•)* and *S (•)* denote the avgpool along frequency, the fully connected block, and the softmax, respectively. After that, the convolution branch applies *k* × *k* convolutions to generate *n* features $U^{Conv}\in R^{n\times T\times F\times C^{’}}$, which are then multiplied with the weights generated by the attention branch and then summed to get the final output $U_{F}^{O}\in R^{T\times F\times C^{’}}$.

(5) $$U^{Conv}=Conv\left(U\right)$$

(6) $$U_{F}^{O}=\sum_{i=1}^{n}{\bar{A}}_{i}^{f}⊗{U_{i}}^{Conv}$$

where *n* and ⊗ denote the number of basis kernels and the multiplication of elements, respectively. The improved TDC is similar to the improved FDC; only the avgpool dimension is different.

(7) $$U^{t}=avg\left(U,F\right)$$

(8) $${\hat{U}}^{t}=ReLU\left(BN\left(Conv\left(U^{t}\right)\right)\right)$$

(9) $$A^{t}=avg\left({\hat{U}}^{t},T\right)$$

(10) $${\bar{A}}^{t}=S\left(FC\left(A^{t}\right)\right)$$

(11) $$U^{Conv}=Conv\left(U\right)$$

(12) $$U_{T}^{O}=\sum_{i=1}^{n}{\bar{A}}_{i}^{t}⊗{U_{i}}^{Conv}$$

### 3.3. Parallel Time-Frequency Multi-Scale Attention (PTFMSA) Module

**Figure 5:**
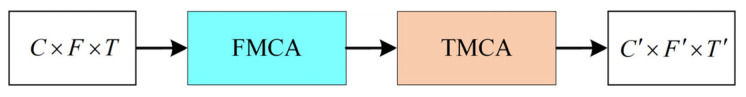
The illustration of a cascade of FMCA and TMCA.

**Figure 6:**
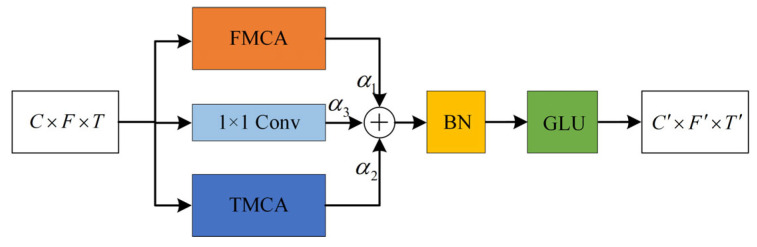
PTFMSA module.

The traditional way of acquiring time-frequency features is to directly cascade two modules [28,29] as shown in Figure 5. However, the cascade approach has the disadvantage that temporal features and frequency features may interfere with each other. When temporal features are extracted, the network focuses more on the correlation between different time frames and suppresses the importance of different frequency bands. When frequency features are extracted, the network will focus more on the importance of different frequency bands and ignore the correlation between different time frames. Therefore, we propose the parallel time-frequency multi-scale attention (PTFMSA) module that uses the structure of parallel branches along the time dimension and frequency dimension to avoid interference between features of different dimensions as shown in Figure 6. The main purpose of the 1 × 1 Conv branch is to change the number of channels of the input feature so that they can be added together. The important coefficients of different branches are also put into the network for training to ensure optimal coefficients. We can express it as

(13) $$U^{’}=\alpha_{1}U_{\mathrm{FMSA}}+\alpha_{2}U_{\mathrm{TMSA}}+\alpha_{3}U_{\mathrm{conv}}$$

(14) $$\alpha_{1}+\alpha_{2}+\alpha_{3}=1$$

where $\alpha_{1}$, $\alpha_{2}$, $\alpha_{3}$ are learnable dynamic parameters with the same initial values of 1/3, $U_{\mathrm{FMSA}}$ is the feature of the FMSA branch outputs, $U_{\mathrm{TMSA}}$ is the feature of the TMSA branch outputs, $U_{\mathrm{conv}}$ is the feature of the 1 × 1 Conv branch outputs, and $U^{’}$ is the feature of the sum of the three branching features.

Experimental results show that learnable dynamic parameters that can dynamically adjust the weights of different branches during network training can perform better compared to when they are all set to a fixed size of 1/3. After the BN layer, we apply the GLU layer to replace the ReLU layer, which can help the network capture long-term dependencies and contextual information.

**Figure 7:**
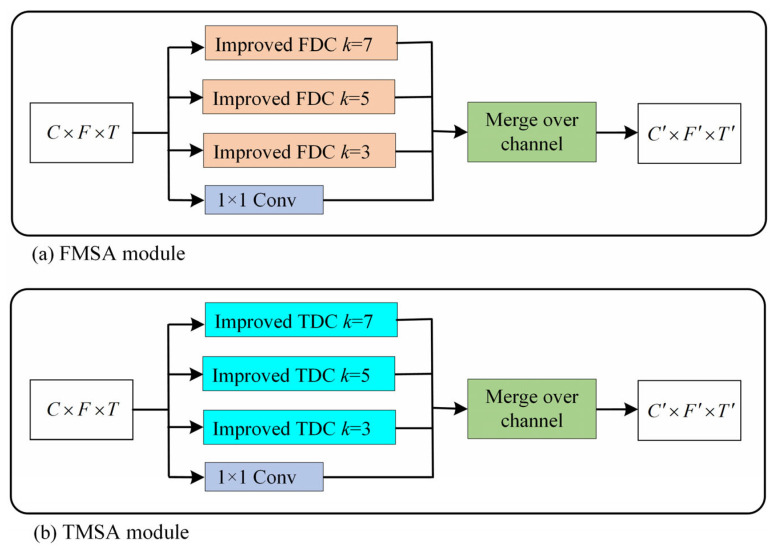
FMSA module and TMSA module.

As shown in Figure 7a,b, we propose a frequency multi-scale attention (FMSA) module and a temporal multi-scale attention (TMSA) module that apply improved dynamic convolution for feature extraction at multiple scales in the frequency and time dimensions, respectively. This enables the features containing more information to avoid the limitations of the convolutional feature and ensure physical consistency. For improved FDC, the number of basis kernels affects the performance of the module and the speed of convergence. The optimal number of basis kernels was obtained through experimental comparison by setting different numbers of base kernels. In this way, we set *k* to be 3, 5, and 7, corresponding to three different scales of improved FDCs, respectively. These improved FDCs were applied to the input features to obtain features of different receptive fields. For the improved FDC with *k* set to 1, a convolution with kernel size 1 is used to replace it, which can reduce the amount of computation and avoid the problem of gradient disappearance or gradient explosion. In order to prevent different scale features from interfering with each other, we merge rather than add these four scale features in the channel dimension. Based on the above design, the FMSA module not only enriches the receptive field of features but also enforces frequency-dependency on 2D convolution. The temporal multi-scale attention (TMSA) module is an implementation of the multi-scale attention module along the frequency dimension, as shown in Figure 7b. It is designed along the same lines as the FMSA module, except that the dimension has been changed from frequency to time, which can strengthen the connection and importance of time frames at different scales.

## 4. Experiments

### 4.1. Dataset and Evaluation Metrics

The ESC-50 [23] dataset contains 2000 environmental audio recordings and is commonly used for benchmarking ESC tasks. Each sample is a standard 5 s recording with a sampling rate of 44.1 kHz. It is divided into five major categories, and each major category is further subdivided into 10 categories as given in Table 1. Therefore, there are 50 categories in total, and each category contains 40 audio samples.

For the performance of all networks in the dataset, we use the classification accuracy as our evaluation metric in Equation (15). Classification accuracy is one of the most intuitive and commonly used metrics for ESC tasks.

(15) $$Classification\;Accuracy=\frac{TP+TN}{TP+FP+TN+FN}$$

where TP is true positive, TN is true negative, FP is a false positive, and FN is false negative.

This dataset has been evenly divided into five sets, with one set selected as the validation set each time. The remaining four sets are trained using BC learning to generate 2000 training set samples for five-fold cross-validation, with average accuracy as the metric.

### 4.2. Training Details

The raw audio was down-sampled to 20 kHz, and the portion between the first non-zero point to the last non-zero point was retained. To make the training more effective, we use BC learning [30] to mix the dataset, which can enlarge Fisher’s criterion in the feature space and make the positional relationship of feature divisions more obvious. We randomly select two clips from two different classes and then mix them as input to the network to train the corresponding mixing ratio. The size of the clip is set to 30,225 (about 1.51 s audio). The mixing formula is as follows:

(16) $$mix_{s}\left(s_{1},s_{2}\right)=\frac{ps_{1}+\left(1-p\right)s_{2}}{\sqrt{p^{2}+{\left(1-p\right)}^{2}}}$$

(17) $$p=\frac{1}{1+10^{\frac{G_{1}-G_{2}}{20}}\times \frac{1-r}{r}}$$

where $s_{1}$ and $s_{2}$ are randomly selected clips from two different classes, $G_{1}$ and $G_{2}$ are the maximum gains of the clips $s_{1}$ and $s_{2}$, and r is a random number between 0 and 1.

**Figure 8:**
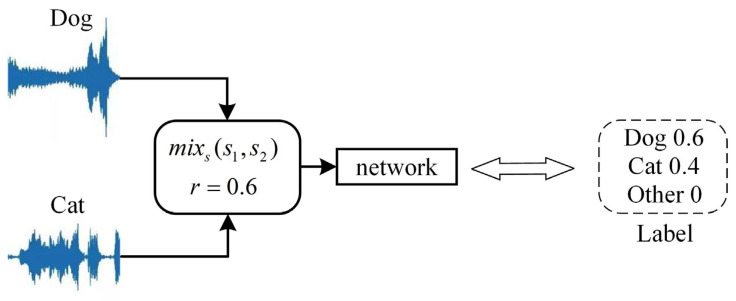
The mixing process of BC learning.

For example, if $s_{1}$ and $s_{2}$ randomly selected dog and cat, the mixing process of BC learning is shown in Figure 8.

The number of mixed datasets generated is 2000, as before. The training will terminate after 2000 epochs, and the learning rate is initially set to 0.1 and then changes to one-tenth of the previous value every 600 epochs. The loss function is chosen to be KLDivLoss [30] and Stochastic Gradient Descent is used as the optimizer. The formula for KLDivLoss is as follows:

(18) $$\begin{array}{c} L=\frac{1}{n}\sum_{i=1}^{n}D_{KL}\left(y^{\left(i\right)}∥f_{\theta }\left(x^{\left(i\right)}\right)\right)\\ \;\;\;\;\;\;\;\;\;=\frac{1}{n}\sum_{i=1}^{n}\;\sum_{j=1}^{m}\;y_{j}^{\left(i\right)}\log\frac{y_{j}^{\left(i\right)}}{{\left\{f_{\theta }\left(x^{\left(i\right)}\right)\right\}}_{j}} \end{array}$$

(19) $$\theta \leftarrow \theta -\eta \frac{\partial L}{\partial \theta }$$

where x is the input, y is the label, $f_{\theta }$ is the approximation, n is the batch size, m is the number of classes and η is the learning rate.

### 4.3. Test Details

**Figure 9:**
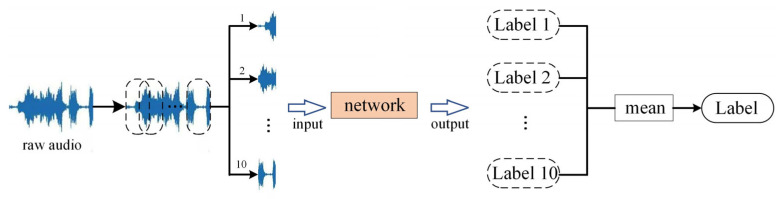
The test process of a single audio.

After the introduction of BC learning, the test result for a single audio is the average of its 10 clips’ predictions as shown in Figure 9. The input raw audio is divided equally into 10 clips of length 30,225. Then, 10 clips are fed into the network to output the corresponding labels and take the average of 10 labels as the final label for the audio.

## 5. Results and Discussion

### 5.1. Optimal Number of Layers for the PTFMSAN

**Figure 10:**
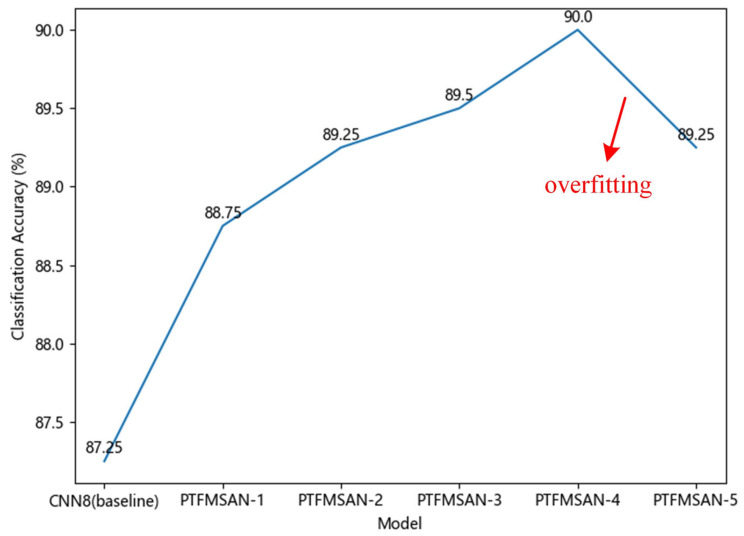
Classification accuracy trends of 5 different PTFMSAN structures.

A moderate number of network layers will result in the best network performance. If the number of layers is too small, it will lead to the network being too simple to learn comprehensive features. If there are too many layers in the network, it will lead to the network being too complex, resulting in overfitting. Therefore, it is necessary to choose the appropriate number of PTFMSA layers. CNN8 is used as a baseline model, which consists of four blocks. Each block is composed of two 2D convolutional layers with a kernel size of 3 and a maxpool layer. All convolution layers are followed by a BN and a ReLU layer by default. We changed the number of PTFMSA layers and designed five PTFMSANs of different depths to explore the best network structure as our final PTFMSAN. Table 2 demonstrates the comparison results of five different PTFMSAN structures on the ESC-50. From Figure 10, we can visually see that PTFMSAN-1, PTFMSAN-2, PTFMSAN-3, PTFMSAN-4, and PTFMSAN-5 all outperformed the baseline network, and as the number of PTFMSA layers increases the performance of the network gradually improves. Compared with PTFMSAN-4, PTFMSAN-5 has poorer performance, indicating the onset of overfitting. From this, PTFMSAN-4 is chosen as our final HLFE configuration of the PTFMSAN architecture.

### 5.2. Comparison with State-of-the-Art Models

To estimate the performance of PTFMSAN, we compare it with some state-of-the-art CNN-based models on the ESC-50 dataset, including [16,23,30,31,32,33,34,35,36]. Table 3 shows the comparison between the performance of our proposed network and state-of-the-art CNN-based models, including time-domain signal input and frequency-domain signal input. It can be seen that our proposed network has few parameters and achieves a high classification accuracy of 90% on the ESC-50 dataset, which proves that the proposed PTFMSAN can effectively extract time-frequency features of sound in the ESC task. The proposed structure provides an efficient time-frequency feature extraction method for the ESC task. However, its generalization ability has to be further verified.

### 5.3. Ablation Experiments

The results in the previous subsections show that the proposed network performs better compared to other state-of-the-art CNN-based models. In order to prove the effectiveness of each module, we conducted ablation experiments. CNN8-DC denotes the replacement of each layer in the HLFE module of CNN8 by a conventional dynamic convolution, and other networks are similarly replaced.

#### 5.3.1. The Effectiveness of Improved Dynamic Convolution

Compared to traditional dynamic convolution (DC) using only fully connected layers and FDC, this paper combines convolution and fully connected layers to extract global and local features to obtain better results. To verify that the improved dynamic convolution is superior to other dynamic convolutions, we conduct experimental comparisons between the improved dynamic convolution and other dynamic convolutions. In Table 4, CNN8-DC denotes the replacement of each layer in the HLFE module of CNN8 by a conventional dynamic convolution, and other networks are similarly replaced. The results show that the enhanced model performs better than both traditional DC and FDC, suggesting that convolution and fully connected layers work well together to capture local and global data.

As can be seen from Table 4, the performance is improved after applying DC and FDC to the CNN8 model. After further introducing our proposed improved dynamic convolution, CNN8-improved FDC and CNN8-improved TDC perform better than CNN8-FDC and CNN8-TDC, respectively. This is a good indication that our proposed improved dynamic convolution not only ensures shift-invariance of the 2D convolution along the frequency dimension but also avoids the loss of global features. In addition, CNN8-TDC performs worse than the baseline model, but after improvement, it exceeds the baseline model.

#### 5.3.2. The Effectiveness of the Multi-Scale Attention Module

This paper applies improved dynamic convolution for feature extraction at multiple scales and merges in the channel dimension to enrich the receptive field of features. In order to verify the effectiveness of the multi-scale attention module, we designed models for DC, FDC, TDC, improved FDC, and improved TDC at single and multiple scales and conducted experimental comparisons.

**Figure 11:**
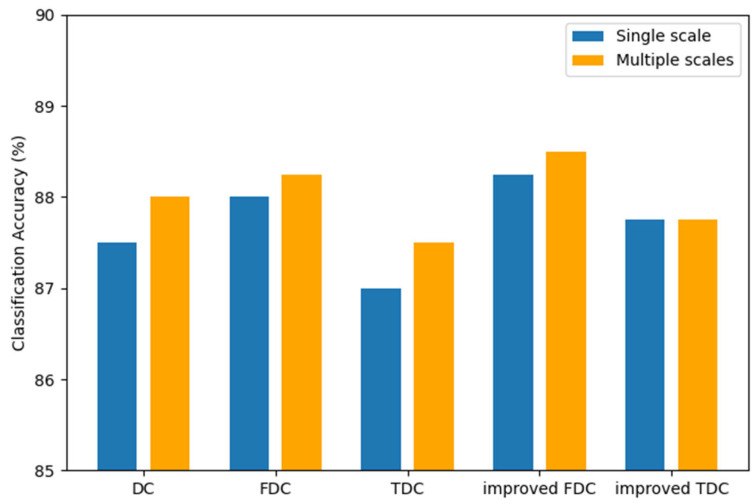
Classification accuracy of dynamic convolutions at single and multiple scales.

As shown in Table 5 and Figure 11, compared to single-scale dynamic convolution, multi-scale dynamic convolution generally performs better. This can enrich the receptive field of features and makes the features contain more information to avoid the limitations of the convolutional feature. It is clear that multi-scale attention modules are effective. In addition, the proposed improved FDC and improved TDC still outperform FDC and TDC at multiple scales, which also indicate that the improved dynamic convolutions are more compatible with multiple scales.

#### 5.3.3. The Effectiveness of Parallel Branch Structure

**Figure 12:**
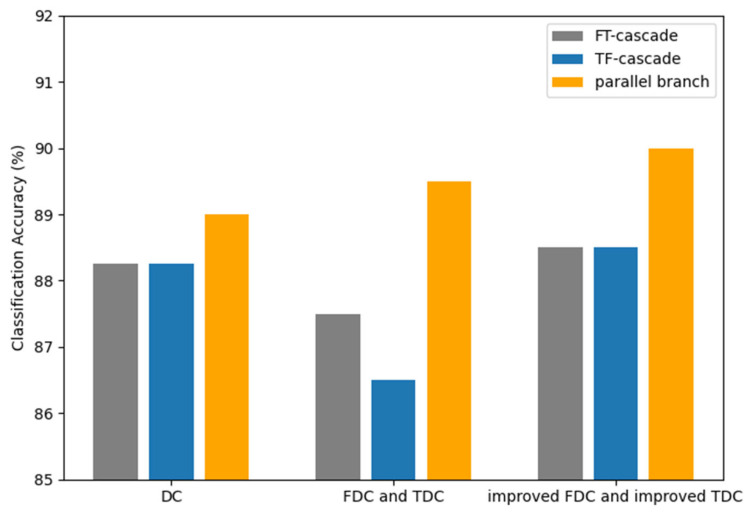
Classification accuracy of cascade and parallel branch structure.

The proposed PTFMSA module applies a parallel branch structure to avoid interference between features of the time domain and the frequency domain. In order to verify the effectiveness of the parallel branch structure, we design a comparison experiment between the cascade structure and the parallel branch structure. In Table 6 and Figure 12, FT-cascade denotes a cascade structure for extracting frequency domain features followed by time domain features, and TF-cascade denotes a cascade structure for extracting time domain features followed by frequency domain features.

As shown in Table 6 and Figure 12, the comparison shows that the parallel time-frequency design avoids mutual interference between features, making it more effective than the cascade structure. This is a good indication of the effectiveness of the parallel branch structure.

#### 5.3.4. The Effectiveness of GLU and Learnable Dynamic Parameters

**Figure 13:**
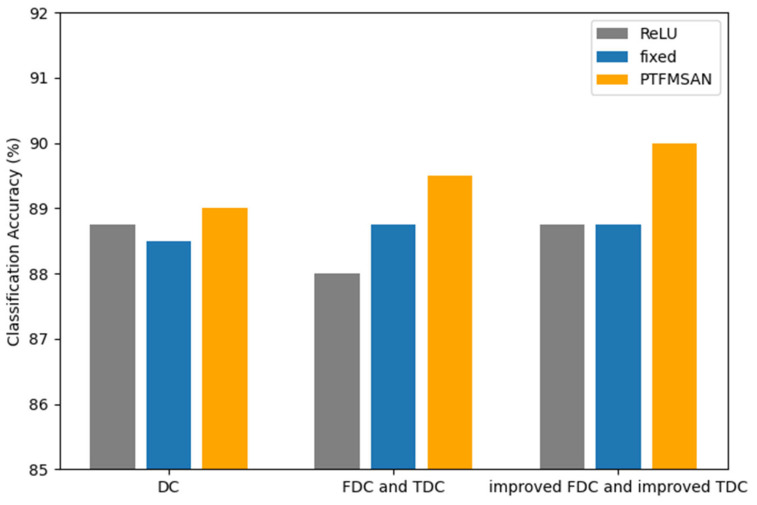
Classification accuracy of different activation functions and branch weights.

The proposed PTFMSA module applies the GLU activation function instead of the traditional ReLU activation function and uses learnable dynamic parameters to adjust the weights of the different branches. In order to verify their effectiveness, we design a comparison experiment. In Table 7 and Figure 13, fixed denotes the parameters α_1_, α_2_, α_3_, which in Equation (14) are all set to a fixed value of 1/3 instead of participating in the network training, and ReLU denotes the model that replaces the GLU of the PTFMSA module with the ReLU activation layer.

As we can see in Table 7 and Figure 13, both the GLU activation function and the learnable dynamic parameters can improve the performance of the network. This proves that the GLU activation function can effectively help the network better capture long-term dependencies in sequential data, and learnable dynamic parameters can dynamically adjust the weights of different branches during network training.

Overall, the ablation experiments verify that every suggested element adds to the ultimate precision of PTFMSAN.

Improved dynamic convolution, multi-scale kernels, a parallel time-frequency architecture, and adaptive gating/branch weights together explain the superior results. Importantly, the model remains compact, with just about 3.7 million parameters (about 14.8 MB in FP32), demonstrating its suitability for implementation on low-end hardware.

### 5.4. The Optimal Number of Basis Kernels

As mentioned earlier, the number of basis kernels affects the performance and convergence speed of the module, so it is necessary to obtain an appropriate number of basis kernels. The experiment is set up to solve this problem.

The number of basis kernels was set to the most commonly used 3, 4, and 5 for experimental comparison on various improved network structures based on CNN8. As shown in Table 8, under the same experimental conditions, incomplete feature extraction in different dimensions results in the loss of some features, while an excessive number of basis kernels can overfit noise and reduces generalization ability.

We observe that cross-dataset evalution (such as UrbanSound8K or AudioSet subsets) is useful even though ESC-50 is still a commonly used standalone benchmark for architectural studies in environmental sound classification [16,25,30,33,34]. We leave this as a viable area for future research to evaluate the suggested model’s generalizability and robustness.

## 6. Conclusions

In this paper, we improved dynamic convolution by combining convolution and fully connected layers to extract global and local features, ensuring shift-invariance of 2D convolution in the frequency dimension. The proposed PTFMSA module applies improved dynamic convolution for feature extraction at multiple scales, and the introduction of parallel branches avoids the mutual interference of time-frequency features. Based on the PTFMSA module, we propose PTFMSAN for ESC tasks. The results show that our proposed network achieves improvements in ESC tasks and has a high classification accuracy of 90%, competitive among CNN-based networks. While recent Transformer-based models have achieved higher absolute accuracy on ESC-50 with large-scale pre-training, our result demonstrates that compact CNN-based architectures remain effective and competitive. In addition, ablation experiments prove the effectiveness of our proposed modules. The proposed structure provides an efficient time-frequency feature extraction method for the ESC task. On limited hardware platforms, PTFMSAN’s high performance and lightweight design allow for effective training and real-world implementation. However, its generalization ability has to be further verified. In future work, we will try to apply it to other fields to verify its generalization ability. Meanwhile, we will try more attention module methods to improve the classification accuracy.

## Figures and Tables

**Table 1:** Five major categories and the name of each category in the ESC-50.

Animals	Natural Sound Spaces & Water Sounds	Human, Non-Speech Sounds	Interior/Domestic Sounds	Exterior/Urban Noises
Dog	Rain	Crying baby	Door knock	Helicopter
Rooster	Sea waves	Sneezing	Mouse click	Chainsaw
Pig	Crackling fire	Clapping	Keyboard typing	Siren
Cow	Crickets	Breathing	Door, wood creaks	Car horn
Frog	Chirping birds	Coughing	Can opening	Engine
Cat	Water drops	Footsteps	Washing machine	Train
Hen	Wind	Laughing	Vacuum cleaner	Church bells
Insects (flying)	Pouring water	Brushing teeth	Clock alarm	Airplane
Sheep	Toilet flush	Snoring	Clock tick	Fireworks
Crow	Thunderstorm	Drinking, sipping	Glass breaking	Hand saw

**Table 2:** The comparison results of 5 different PTFMSAN structures on the ESC-50.

Model	Classification Accuracy
CNN8 (baseline)	87.25%
PTFMSAN-1	88.75%
PTFMSAN-2	89.25%
PTFMSAN-3	89.50%
PTFMSAN-4	90.00%
PTFMSAN-5	89.25%

**Table 3:** The comparison results with state-of-the-art CNN-based models on the ESC-50 dataset. Note: “PTFMSAN” refers to the four-layer variant (PTFMSAN-4).

Model	Classification Accuracy	Parameters
EnvNet-v2 + BC learning [29]	81.80%	18.0 M
AemNet-DW (Scratch) [30]	76.80%	3.0 M
LGTFB-EN-CNN [31]	86.20%	-
MSARN [32]	81.60%	3.1 M
M-LM-C CNN [33]	85.60%	11.3 M
TSCNN [34]	87.30%	-
Mixup augmentation [22]	83.90%	-
CNN + Fusion Global [35]	88.65%	-
PTFMSAN (ours)	90.00%	3.7 M

**Table 4:** The performance of the improved dynamic convolution and other dynamic convolutions.

Model	Classification Accuracy
CNN8 (baseline)	87.25%
CNN8-DC	87.50%
CNN8-FDC	88.00%
CNN8-TDC	87.00%
CNN8-improved FDC	88.25%
CNN8-improved TDC	87.75%

**Table 5:** The performance of dynamic convolutions at single and multiple scales.

	Classification Accuracy (%)	
	Single Scale	Multiple Scales
DC	87.50%	88.00%
FDC	88.00%	88.25%
TDC	87.00%	87.50%
improved FDC	88.25%	88.50%
improved TDC	87.75%	87.75%

**Table 6:** The performance of the cascade and parallel branch structure.

	Classification Accuracy		
	FT-Cascade	TF-Cascade	Parallel Branch
DC	88.25%	88.25%	89.00%
FDC and TDC	87.50%	86.50%	89.50%
improved FDC and improved TDC	88.50%	88.50%	90.00%

**Table 7:** The performance of different activation functions and branch weights.

	Classification Accuracy		
	ReLU	Fixed	PTFMSAN
DC	88.75%	88.50%	89.00%
FDC and TDC	88.00%	88.75%	89.50%
improved FDC and improved TDC	88.75%	88.75%	90.00%

**Table 8:** The performance of different numbers of basis kernels on various improved networks. The results show that four basis kernels provide the best trade-off between accuracy and generalization.

	The Number of Basis Kernels		
	3	4	5
DC	85.00%	88.25%	85.00%
DC-fixed	86.00%	87.25%	85.75%
FDC	84.75%	88.00%	87.75%
TDC	84.00%	87.00%	87.00%
FDC-TDC-parallel	84.50%	89.50%	89.50%
FMSA	83.75%	88.25%	86.75%
TMSA	82.75%	87.50%	87.00%
MSA-FT	82.75%	87.50%	82.75%
MSA-TF	81.25%	86.50%	84.00%
PTFMSAN-1	86.00%	88.75%	87.25%
PTFMSAN-2	85.50%	89.25%	84.50%
PTFMSAN-3	86.00%	89.50%	86.00%
PTFMSAN-4	84.50%	90.00%	84.75%
PTFMSAN-5	85.50%	89.25%	84.50%
PTFMSAN-fixed	85.00%	88.75%	86.50%
PTFMSAN-ReLU	86.75%	88.00%	86.50%
improved FDC	83.75%	88.25%	81.50%
improved TDC	83.75%	87.75%	81.75%
improved FMSA	84.50%	88.50%	85.50%
improved TMSA	85.00%	87.75%	85.75%
improved MSA-FT	85.25%	88.50%	85.50%
improved MSA-TF	85.00%	88.50%	85.75%

## Data Availability

The datasets generated and analyzed during the current study are available in the karolpiczak repository, https://github.com/karolpiczak/ESC-50, accessed on 30 September 2023.

## Abbreviations

CNN	Convolutional neural networks
ESC	Environmental sound classification
PTFMSAN	Parallel time-frequency multi-scale attention network
BC	Between-class
DL	Deep learning
MFCC	Mel-scale frequency cepstral coefficients
RNN	Recurrent neural networks
FDC	Frequency dynamic convolution
FMSA	Frequency multi-scale attention
TMSA	Temporal multi-scale attention
PTFMSA	Parallel time-frequency multi-scale attention
TFC	Time-frequency conversion
HLFE	High-level feature extraction
BN	batch normalization
ReLU	Rectified Linear Unit
TDC	Temporal dynamic convolution
DC	Dynamic convolution
